# Kinect and Few-Shot Technology-Based Simulation of Physical Fitness and Health Training Model for Basketball Players in Plateau Area

**DOI:** 10.1155/2022/2256522

**Published:** 2022-04-11

**Authors:** Xijun Hong

**Affiliations:** Teaching and Research Section of Basketball, College of Sports Science, Dali University, Dali 671003, China

## Abstract

Players in modern basketball have a lot of physical contact, a lot of bumps, and a lot of physical struggles. The competition for the ball, whether in the air or on the ground, is fierce, putting higher demands on the players' physical abilities. Coaches frequently use plateau physical training, which is very effective in developing athletes' cardiopulmonary function, among many other training methods. The proportional length and active area of arms are obtained using the skin color model of the human body, the angle and posture information of each joint is extracted from dynamics, and the 3D posture of arms and dynamic arms is trained and recognized in this paper, which is based on Kinect. The findings revealed that mild hypoxia in the plateau significantly lowered basketball players' performance and that basketball players' maximum heart rate and 1-minute heart rate recovery in high-intensity exercise were lower than those in flat area training.

## 1. Introduction

Altitude training refers to a special training method that uses the double stimulation of altitude hypoxia and exercise hypoxia to deepen the stress reaction of the body and improve the various functions and sports ability of the body [1]. Basketball is a combination of walking, sports, and running at various speeds based on the anaerobic decomposition of high-energy phosphate compounds, which is characterized by the mixture of anaerobic metabolism and aerobic metabolism [2]. Athletes' physical examinations are an important part of their physical development, and they help to promote their physical development to some extent. The collection, collation, and analysis of training data have become an important link with continuous scientific training [3, 4], which is the main reason for including physical training in the basketball training plan, improving the physical fitness of basketball players to a certain level, ensuring the improvement of athletes' physical fitness, and establishing a certain physical reserve in competition to ensure the use of more effective tactics and commands [5].

With the rapid development of the intelligent industry, the application scope of robots in various industries is constantly expanding, and artificial intelligence technology [6–8] has penetrated into people's work and life. 3D gesture recognition in dynamic arm is an important way to improve the technical level of artificial intelligence, and it is also a major problem to be solved urgently in the field of intelligent technology. The use of RGB-D sensors like the Microsoft Kinect is a convenient and effective way to obtain a diverse and realistic 3D human model, but existing methods of extracting whole body point cloud information for 3D modeling are complicated in terms of operation, equipment, and subsequent processing, and the modeling effect is not suitable for 3D crowd animation [9]. With the introduction of Kinect technology, a new type of disease rehabilitation system has emerged, allowing athletes to select the appropriate exercise mode based on their current situation, allowing for more targeted and planned rehabilitation and a desire to recover as quickly as possible [10]. The new rehabilitation exercise system adds a variety of exercise methods, allowing players to customize their workouts based on their preferences and improving their real-world experience. The new system can also help doctors make more accurate and scientific exercise plans, reduce workload, improve the efficiency of doctors' diagnosis and treatment, and help athletes achieve faster health recovery.

Although the recent research on plateau training has made some achievements, due to the different characteristics of each event, there is no plateau training model suitable for all sports and athletes [11], which has great theoretical and practical value. Kinect equipment has achieved interdisciplinary and has a good development prospect and economic value. After the introduction of Kinect technology, the overall functions of the training system can become more powerful and rich. When athletes use the training system, they can choose their own training mode in a targeted way and get the best results with proper exercise. In order to scientifically explore the high-level physical training of high-level basketball players, the changes of body function and body shape before and after the plateau were deeply studied.

## 2. Related Work

Some basketball players have competed in terms of training quantity and intensity, and their mutual cooperation has been strained. In general, there is a negative correlation between basketball players and their training. That is, as the training amount increases, the training intensity decreases [12, 13], and as the training intensity increases, the training amount decreases as well [12, 13]. The difference between basketball and other sports, according to literature [14], is that it is the same game in which space, ground, time, and speed compete with each other around the basket in the air. Basketball is a speed-strength, confrontation-endurance-skill event aimed at fast and changeable attack and defense [15]. According to literature [16], anaerobic metabolism and anaerobic and aerobic mixed metabolism are the primary sources of energy for elite female basketball players in competition, with aerobic metabolism and aerobic metabolism as backups. According to literature [17], short-term actions in basketball games such as fast layups, jumping, shooting, dribbling, and rebounding are primarily dependent on energy conversion from ATP (adenosine triphosphate) to CP (creatine phosphate), but this energy supply is not limited to 10–15 seconds and is primarily triggered by the system during continuous attack and defense conversion. Basketball players should have tall, well-proportioned, slender limbs; big hands; and big feet [18]. Other related works' qualitative descriptions of basketball players' physical characteristics are mostly similar to those mentioned above, so I will not repeat them here.

Nowadays, Kinect technology researched and designed by Microsoft Corporation is more and more used in medical care. Reference [19], in conjunction with Kinect technology and system, proposes a system for assisting upper-limb recovery in stroke athletes that has shown to be effective. Reference [20] has conducted professional research on the rehabilitation assistant robot, focusing on the intensity control of the robot and the training scheme implementation. Literature [21] has created a new type of equipment that uses Kinect technology to obtain athletes' bone data, creates the best recovery training plan for athletes, and monitors their training results and disease recovery. The factors affecting the random error of depth data when using Kinect are discussed in [22]. Reference [23] proposes a method for reconstructing a 3D model of the human body that involves using 24 Kinect devices to collect registration and fusion of local point clouds from various parts of the body, followed by template fitting. Reference [24] proposes a method to measure human morphological parameters by using the depth information of the front and side of human body obtained by the second generation Kinect equipment and creates a new model by modifying the standard human body model. In [25], RGB-D sensor based on Kinect extracts the information of human point cloud from 6 directions, 3 pitch angles, and a total of 18 viewing angles and uses it in 3D human reconstruction method combined with improved point cloud alignment algorithm. Reference [26] proposes a 3D gesture recognition method of dynamic arm based on D-S evidence theory algorithm, which uses gesture sensor to acquire and analyze 3D gesture information from human dynamic arm. Reference [27] puts forward a 3D gesture recognition method of dynamic arm based on classification feature extraction. The background model of dynamic arm is built based on adaptive Gaussian mixture model, and the human arm region is divided by fusion background subtraction method. The limited scanning accuracy and range of Kinect equipment limit the accuracy of 3D mannequin to a certain extent. Multiple devices or multiple small scans are used to improve the scanning accuracy and reduce the scanning range. This will lead to hardware environment, operation methods, and problems. The algorithm is very complicated to implement.

Indoor strength training, aerobic endurance training on land, exercises after strength conversion, and coordination and relaxation exercises make up the majority of the regular physical education class, which lasts about 2 hours. Prior to technical training, 40–60 minutes of special preparation activities is required. According to the literature [28], the 5000 m and 10000 m swimming events are 8% lower than sea level, and the 100 m swimming events are 2–3% lower, with the decline becoming more apparent as the movement distance increases [28]. Working at a 2000 m altitude increased the heart rate by 10–20% when compared to working on the plain; according to literature [29, 30], when the altitude is 1000 meters or higher, the maximum oxygen uptake drops by 10.5 percent. The decrease in VO_2_ max was linked to an increase in oxygen tolerance as altitude was increased, according to research. Blood flow and oxygen saturation are also involved. Basketball players can benefit from altitude training not only in terms of aerobic endurance, but also in terms of lactic acid energy supply. As a result, team sports like basketball require altitude physical training to enhance athletes' physical fitness during the pre-match preparation stages.

## 3. Research Method

### 3.1. Research Design

#### 3.1.1. Research Objects

All the players took part in and completed the test. The average age of the subjects was 26.5 years, average body weight 70.4 kg, and average height 174.8 cm.

#### 3.1.2. Literature Data Method

Through China Journal Network and National Library, a lot of literature on basketball selection, physiology, physical education, and modern training theory was retrieved and collected, basically focusing on the research and development of basketball in China. By reading and classifying various papers, relevant data were analyzed, classified, and synthesized according to the physical characteristics of basketball players.

#### 3.1.3. Expert Interview Method

I had the opportunity to take part in the physical fitness test of Chinese basketball players in winter training and interviewed five coaches according to the prepared interview outline, so as to understand the specific situation of physical fitness training of Chinese basketball players and do more in-depth research.

#### 3.1.4. Test Method

The complete test lasted two months and was divided into two parts. All subjects underwent elevation and routine electrocardiogram (ECG) before the experiment, and all subjects' ECGs were normal. During the whole testing process of the two sites, ECG samples of the subjects were obtained and no abnormal ECGs were found. Physical function test finger blood was collected on an empty stomach at around 7:00 a.m., and body composition test was conducted at around 7:00 a.m. to complete the tester analysis.

### 3.2. Physical Training Process

Fitness should be defined as the combination of various physical abilities required of athletes in order to maximize the flexibility of various organs and systems of the body, overcome fatigue, and produce exceptional performance under the stress of special training and competition. Physical and intangible physical strength are included. Physical ability is referred to as tangible physical ability, while psychological ability is referred to as intangible physical ability. Physical quality is divided into three categories: physical structure, physical function, and mental willpower. There are two types of stretching: static and dynamic. Basketball is a type of high-intensity anaerobic exercise that causes muscles to produce a lot of lactic acid. As a result, athletes should do warm-up and relaxation exercises to improve their health and performance in sports. The comprehensive principle, ladder principle, goal principle, proper subordinate principle, and recovery principle should be followed when developing a basketball plan.

Physical training at altitude is a well-thought-out method. It is a good pre-competition training method for improving athletes' performance by assembling athletes in a large enough area and conducting routine special sports training under anoxia and anoxia conditions. Altitude training helps to stimulate and unlock the body's full potential, allowing the body's absorption, transportation, and utilization of oxygen to be improved and enhanced, and thus the body's functions and athletic performance to be maximized. As a result, sports performance in training and competition will improve.

**Figure 1:**
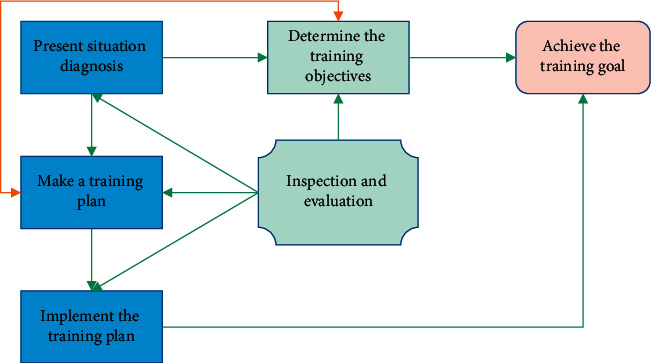
Basic structure of exercise training process.

Physical training is the process of transferring athletes' physical strength from the actual state to the target state. According to the training cycle, the whole physical education process from training to retirement can be regarded as a complete process, which is linked to multiple stages, such as multiyear training courses, annual training courses, and step-by-step training. Every stage of the training process, regardless of the duration, should theoretically cover the basic knowledge: diagnosing the situation of athletes (or sports teams), determining the training objectives, making training plans, and implementing and checking the training plans. The results of the performance evaluation can be found in Figure 1.

In the training process, it can be seen that there are four key steps to effectively change the physical fitness from a relatively chaotic reality state to a relatively orderly target state, realize the effective coordination of all components of physical fitness, accurately grasp the actual situation of athletes (or sports teams), scientifically determine the training objectives, make a careful training plan, ensure the effective implementation of the training plan, and establish a perfect training process monitoring system.

For the training participants, the training goal describes the ultimate goal of the sports training process, and all training activities aid in achieving this ultimate goal. By ensuring that all links and training activities in the training process can be fully developed around the realization of the target state [12], the determination of this ultimate goal provides a basis for the formulation and implementation of training plans and competition plans in the training process. Physical fitness goals, on the other hand, are horizontally related to technical and psychological training goals, so adjustments to other training goals, such as physical fitness requirements, should be taken into account when determining physical fitness goals. Innovation in technology physical training is a subgoal that is made up of several separate goals that must all work together to achieve the desired result.

### 3.3. Kinect-Based Physical Training Model for Basketball Players

Kinect is one of the most popular somatosensory devices on the market. The functional design of this product is mainly to enhance the real experience of gamers in the game. Community interaction is also a key function of Kinect, allowing people to play games together and communicate with others instantly through voice and text [17]. Kinect is far beyond the traditional concept of human-computer interaction in function. It does not need to use other devices to capture the user's physical information in sight, manipulate objects in the interface, provide intelligent and flexible human-computer interaction, realize work functions, and have strong human characteristics.

Kinect somatosensory device has many functions such as speech recognition, microphone input, and advanced sensing technology. The sensing technology of Kinect somatosensory device can fully acquire the video and audio images in the field of vision and use this information to identify the user's identity and voice information and track the bones. For users, Kinect can provide unprecedented interactive experience and achieve four goals: first, bone tracking; second, gesture recognition; third, face recognition; and fourth, voice recognition.

**Figure 2:**
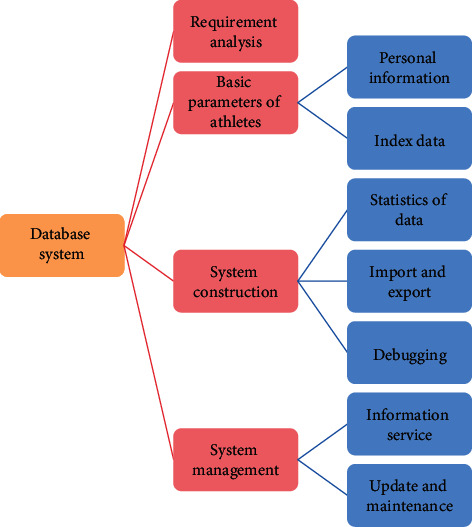
System design process.

Practice shows that database design is software engineering, and the principles, techniques, and methods of software engineering can be applied. Compared with general software engineering, database design has a wider range and is closely related to the application environment, so database design has its own characteristics and training. The database design mainly includes some parts: demand analysis, conceptual structure design, logical structure design and physical structure design, database management, physical fitness test database system design (logical structure design and physical structure design), and database management (Figure 2).

The database of athletes' test data needs to include the functions of summary, input, modification, deletion, storage, download, backup, update, statistical analysis of physical test data, and system security. The system's ease of use is ensured by the integrated friendly operation interface. The DTW (dynamic time warping) method is primarily used to compare two unrelated time series. Different from other methods, when the lengths of two sequences are different or the *X* axis cannot judge them, this method can explain the relationship between the current two sequences by time warping function under certain conditions. In this calculation process, firstly, a matrix of *m* rows and *n* columns should be established, and the distance between points in two sequences should be stored in the matrix to observe the matrix effect. The smaller the value, the higher the similarity between the two sequences. When the calculation reaches the end point, the accumulated value reflects the similarity of the two sequences to a certain extent. The cumulative distance dist(*i*, *j*) can be expressed as the following formula:

(1) $$\begin{array}{c} \text{dist}\left(i,j\right)=\min\left\{\begin{array}{c} \text{dist}\left(i-1,j-1\right)+d\left(A\left(i\right),B\left(j\right)\right),\\ \text{dist}\left(i-1,j\right)+d\left(A\left(i\right),B\left(j\right)\right),\\ \text{dist}\left(i,j-1\right)+d\left(A\left(i\right),B\left(j\right)\right), \end{array}\right) \end{array}$$

where dist(*i*, *j*) represents the cumulative distance, which represents the sum of the distance between data column *A*(*i*) and data column (*j*) plus the cumulative distance of the element closest to the end point.

If each frame of motion data is regarded as a point in 16-dimensional space, then an action sequence can be regarded as a curve in this space. The similarity between two action sequences can be obtained by calculating the distance between the two sets of data, which is usually referred to as Euclidean distance. Then, the Euclidean distance between them is as follows:

(2) $$\begin{array}{c} D\left(M_{1},M_{2}\right)=\sqrt{\sum_{i=0}^{N-1}{\left(G_{i}-G_{i}^{\prime }\right)}^{2}}. \end{array}$$

The smaller the calculation result, the closer the two groups of actions are.

In covariance probability theory and statistics, this parameter represents the total error of variables. Assuming that the two quantities have the same changing trend, they must be both greater than or less than their expected value, in which the calculated covariance is positive. For the same reason, if the two trends are opposite, the calculated covariance is negative.

(3) $$\begin{array}{c} \mathrm{cov}\left(X,Y\right)=E\left(\left(X-\mu \right)\left(Y-\upsilon \right)\right). \end{array}$$

*μ* represents the expectation *E*(*X*) of *X*, and *υ* represents the expectation *E*(*Y*) of *Y*.

The evaluation of rehabilitation training in this system adopts triple evaluation algorithm (DPL algorithm for short), which combines dynamic time warping algorithm, human correlation coefficient, and longest common subsequence algorithm. The data streams of patient motion applying DPL algorithm are sent to DTW algorithm, Pearson correlation coefficient evaluation, and longest common subsequence (LCS) algorithm to obtain the similarity evaluation data with the standard template.

The specific formula of DPL algorithm is as follows:

(4) $$\begin{array}{c} D=\frac{C_{1}}{C_{1}+C_{2}+C_{3}}\times S_{1}+\frac{C_{2}}{C_{1}+C_{2}+C_{3}}\times S_{2}+\frac{C_{3}}{C_{1}+C_{2}+C_{3}}\times S_{3}. \end{array}$$

By setting three confidence variables in advance to a certain extent, the problem that the accuracy of the three fusion algorithms decreases according to the actual situation can be solved, and it can also be adjusted by professional medical staff according to the treatment experience, which is suitable for the rehabilitation of some patients.

In the process of 3D posture recognition in dynamic arm, assuming that *x*,  *y* represent two joint points of the arm, first calculate the difference between the coordinates of the arm and the shoulder node, then judge whether the joint points of each arm are in approximately the same position according to the difference, and then use (5) to calculate the angle (Euclidean distance) between the connection lines of the joint points in dynamic arm:

(5) $$\begin{array}{c} d=K\;\tan\left(Hd_{\text{raw}}+L\right)-O. \end{array}$$

In (5), *d*_raw_ represents the depth value of the three-dimensional posture image in the dynamic arm, and *H*, *L*, *O* represent the constants of the depth value.

According to the distance between the two joint points, calculate the angle between the two joint points of the arm and the *X* axis of the reference point, use the angle of the reference point on the *X* axis to obtain the coordinates of the specified point, and then use (6) to obtain the angular constraint conditions of the arm joint points:

(6) $$\begin{array}{c} P_{A}=\frac{\left\{P_{1},P_{2},\theta ,\tau \right\}}{D\left(X,Y\right)}\times d, \end{array}$$

where *P*_1_ represents the reference point of the arm, *θ* represents the angle between the arm joint point *P*_2_ and the *X* axis, and *τ* represents the set angle threshold.

In the process of three-dimensional posture optimization recognition in dynamic arm, the feature vector sets of angle between arm joint points with different postures are obtained by the following formula:

(7) $$\begin{array}{c} \text{Ktr}=K_{i}\times \frac{\left\{K_{1},K_{2},\ldots ,K_{n}\right\}}{\text{Str}}\text{Str}. \end{array}$$

Among them, {*K*_1_, *K*_2_,…, *K*_*n*_} represents the feature vector of the angle between arm joints in different postures. Str stands for training sample set.

## 4. Result Analysis and Discussion

### 4.1. Physical Training Plan and Load Arrangement

**Figure 3:**
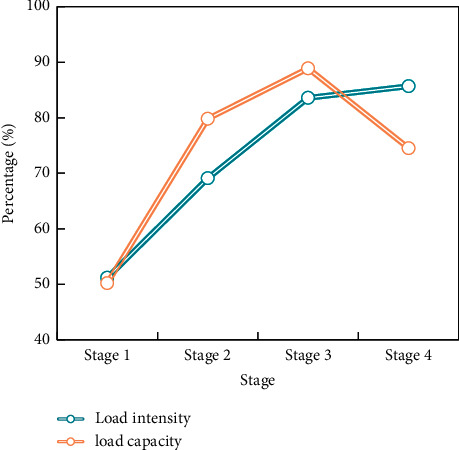
The relationship between altitude train load and intensity change.

Special preparation activities before technical training are also selected according to the content of technical training, mainly including elastic belt shoulder wrist joint maintenance, weight-bearing water bag basketball footwork practice, ball practice, and dynamic stretching. In the definition of load intensity and load quantity in this study, the load intensity is determined as the maximum of the main load indexes (bench push, squat, high pull) in the last two weeks of flat land. The relationship between load intensity and load weight during training is reflected in the percentage of each training index relative to the maximum load value and total training weight (Figure 3).

There is no restricted load applied, the load is only close to the level of flat ground, and the load strength is only about 80% of the maximum strength. A three-day break between two high-intensity strength workouts can help athletes recover completely. The men's basketball team in this study used the plateau to perform strength training, aerobic endurance training, and special skill training, with fat being one of the challenges of this plateau training. Because the waist-hip ratio has changed dramatically, this plateau training can be considered a training goal for body fat reduction.

### 4.2. Analysis of Physical Fitness Test of Basketball Players in Winter Training

Quantitative quality ensures the physical fitness of basketball players and is the material basis for rapidly changing skills and tactics. Modern basketball players need highly developed comprehensive strength. Comprehensive strength is the comprehensive strength of athletes who are engaged in special basketball activities due to the coordination of various sports links. It is the foundation of athletes' special abilities. Improving the overall strength in training is the development trend of modern basketball strength training. In strength training, the training methods of adults are not completely suitable for young athletes, so the physiological characteristics of young athletes must be taken into account in training, based on moderate load, comprehensive and gradual.

**Figure 4:**
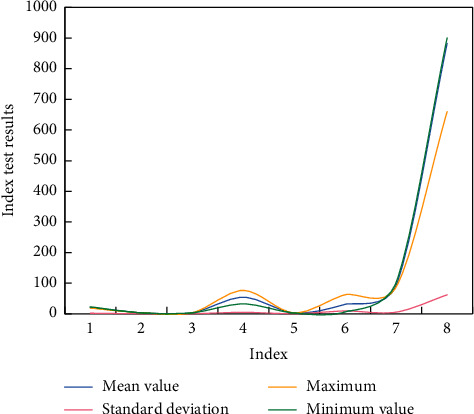
Test and analysis of main physical indexes of Chinese basketball players in winter training.

Figure 4 shows the main physical indexes of young male basketball players in China. Among them, indicators 1∼8, respectively, represent comprehensive running (s), in situ touching height (m), run-up touching height (m), sit-ups, standing long jump (m), 1-minute bench press, turn-back running (s) and 3,200 meters (s).

Generally speaking, the aerobic endurance of Chinese basketball players has obviously decreased, and other indexes have not improved. This shows that the physical training of Chinese youth men's basketball team is not systematic and has not produced good results. Speed endurance and aerobic endurance training can improve other physical qualities of athletes every year, laying the foundation for athletes to reach a higher level.

### 4.3. Comparison of HR Recovery after Exercise in Different Regions

In order to further observe the ability of basketball players to recover cardiovascular function after altitude exercise, the heart rate (HR) recovery at different times after exercise was compared, so as to understand the influence of altitude hypoxia on cardiovascular function. That is, immediately after exercise, the difference between the peak HR of warm-up activity and normal HR is 100%, and the net recovery of HR at different times after two activities in different regions is compared. As a result, the heart rate recovery in 1 minute after high-intensity 10-minute running is obviously faster than that in flat and highland areas, and there is no significant difference between the two places after 4-minute exercise.

**Figure 5:**
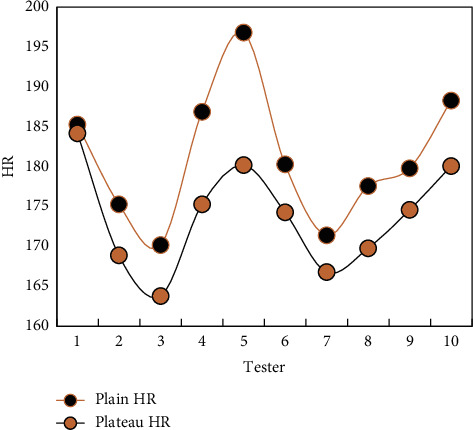
Comparison of HR recovery between plain and plateau immediately after 10 min running.

**Figure 6:**
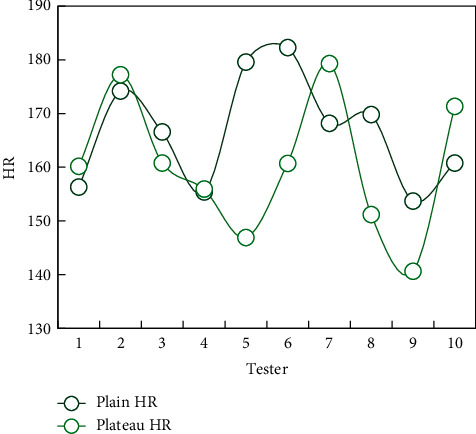
Comparison of 1 min HR recovery after 10 min running.

It can be seen that, even in the 10-minute curve executed in Figures 5 and 6, the change of the individual's normal heart rate is relatively stable. The HR at high altitude is generally higher than normal HR, but individuals with lower HR in plain areas still keep lower HR in high altitude areas.

**Figure 7:**
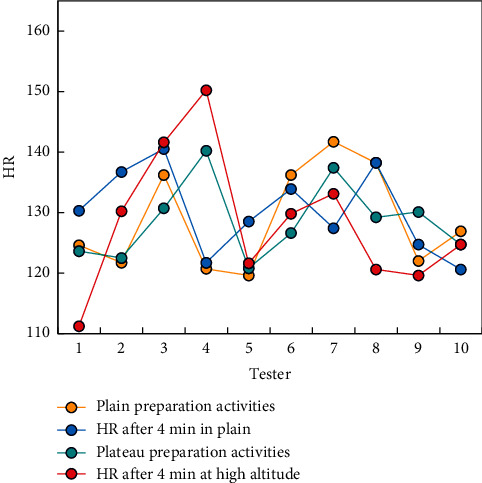
Comparison of 4 min HR after preparation and exercise.

After running for 4 minutes and 10 minutes, HR basically returns to the HR level of the preparation activities, whether in flat or highland areas. It is found that the heart recovery ability of basketball players is not affected by the local altitude (see Figure 7).

The heart is different from other organs in human body. The nutritional system and functional system of different organs are two different systems. The heart not only performs its own functions, but also nourishes the organs themselves. Therefore, insufficient oxygen supply to myocardium is one of the reasons why the heart works at a high level for a long time. On the one hand, increased myocardial contractility raises oxygen consumption and necessitates more blood to perfuse myocardial tissue. Because myocardial tissue relies almost entirely on oxidative metabolism for energy, coronary artery oxygenation is critical [19]. On the other hand, a high HR reduces the diastolic time of the myocardium and makes the coronary artery insufficient in blood supply. Because most coronary artery branches are deeply embedded in the myocardium, the intense compression of myocardial contraction causes a sharp decrease in coronary blood flow.

HR can maintain a relatively stable change in both plain and plateau. Although the overall HR level in the plateau area is higher than that in the plain area, individuals with lower HR in the plain area still maintain a lower HR level during the plateau training period. Therefore, the heart rate can be said to be a very effective index to monitor the changes of cardiovascular function during actual exercise, and it is not affected by the altitude of the area, but only related to exercise intensity, so it can objectively reflect the status of cardiovascular system. During the exercise, the heart deformation and function of athletes of different levels adapt to the training course of specific functions.

### 4.4. Comparison of Algorithm Identification Effectiveness

**Figure 8:**
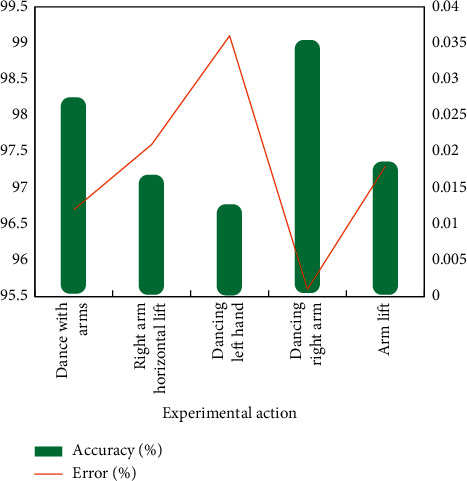
Improve the effectiveness of algorithm identification.

**Figure 9:**
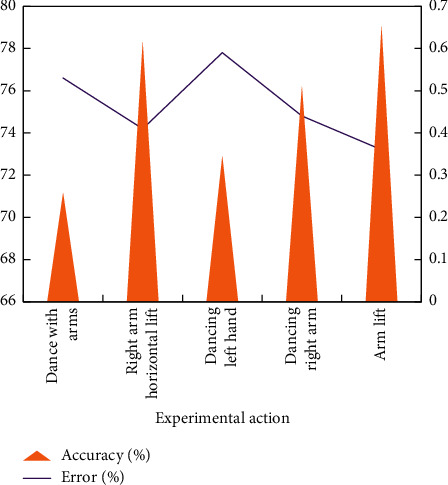
Effectiveness of traditional algorithm identification.

Simulation verification is needed to prove the effectiveness of 3D gesture recognition method in boom based on Kinect. A virtual platform for 3D gesture recognition experiment in boom is built with Windows 7 operating system, and the experimental computer is a personal computer. The number of participants in the experiment was five. Using the improved algorithm and the existing algorithm, 3D postures of dynamic arms were identified, and each person created five kinds of dynamic arm postures. The recognition accuracy and error rate of the two algorithms are compared, and the results are shown in Figures 8 and 9.

The analysis shows that the improved 3D gesture recognition algorithm of dynamic arm has higher accuracy than the existing algorithms and ensures higher accuracy and fewer recognition errors. Using the improved algorithm, in 3D gesture recognition rendering of dynamic arm, the direction, position, and some specific details of human dynamic arm can be well displayed, and the recognition effect is better than that of the existing algorithm, which can effectively meet the requirements.

## 5. Conclusion

Altitude physical training can help basketball players lose weight, but its effect on muscle circumference is dependent on their height, body shape, and training load. Athletes' aerobic metabolism can be improved, allowing them to tap into their full potential and better adapt to high-intensity training and competition. The DTW algorithm is used to evaluate sports, and the DTW distance is calculated as the similarity evaluation index between two sports groups. This method compensates for the traditional Euclidean distance's shortcomings in determining the similarity of nonconformal data. Simulation results show that a 3D gesture recognition method based on the Kinect boom has a high recognition accuracy and can meet intelligent technology application requirements.

## Data Availability

The data used to support the findings of this study are included within the article.
